# Supplementary material for: Association of prior opium addiction with incident non-alcoholic fatty liver disease: A case-control study
Source: PLoS One. 2025 Jul 1;20(7):e0326889. doi: 10.1371/journal.pone.0326889 (PMC12212503; doi:10.1371/journal.pone.0326889)
Supplement: Attachment — Submitted filename: Dear editor of PLOS ONE Journal.docx [file pone.0326889.s001.docx]

**Dear Editor of PLOS ONE Journal,**

First and foremost, I would like to thank the editorial board for the opportunity to review the manuscript entitled “The Impact of Opium Consumption on Non-Alcoholic Fatty Liver Disease: A Case-Control Study” (ID: PONE-D-24-50823). The authors aimed to assess the association between opium consumption and non-alcoholic fatty liver disease, I find the manuscript interesting, However, I have some suggestions for improvement.

**Comments:**

- The term "impact" is typically associated with clinical trials. I recommend changing the title to “Association Between Opium Consumption and Non-Alcoholic Fatty Liver Disease”
- It appears that the study design is a case-cohort design, as the cases derive from a cohort study while controls are selected from the baseline. Additionally, to assess the association between opium and non-alcoholic fatty liver disease, a logistic regression analysis should be conducted. The results must be rewritten based on this new analysis, and if the findings change, the discussion will need to be edited accordingly.
- there are 170 individuals newly diagnosed with NAFLD based on sonography. Therefore, the sample size in the case group should be stated as 170. A low sample size is a significant cause of low statistical power.
- The covariate regarding daily calorie intake ( kilocalories consumed in 24 hours) should clarify how this measurement was obtained.
